# CALR—mutant myeloproliferative neoplasms: insights from next-generation sequencing

**DOI:** 10.1007/s13353-025-00947-7

**Published:** 2025-02-17

**Authors:** Aleksandra Mroczkowska-Bękarciak, Agnieszka Szeremet, Olga Chyrko, Tomasz Wróbel

**Affiliations:** Department and Clinic of Hematology, Cellular Therapies and Internal Medicine, Wroclaw Medical University, Wroclaw, Poland

**Keywords:** Myeloproliferative neoplasm, *CALR* mutation, Next-generation sequencing, Additional mutation

## Abstract

Essential thrombocythemia and primary myelofibrosis belong to the group of *BCR::ABL1*-negative myeloproliferative neoplasms. The presence of mutations in the *JAK2*, *CALR*, and *MPL* genes is essential for the diagnosis of myeloproliferative neoplasms. These mutations are called “driver” mutations. However, not only leading mutations have been identified in patients with MPN, but also more than half of individuals with essential thrombocythemia and more than 80% of patients with myelofibrosis have additional mutations. One technique that makes it possible to find prognostic, predictive, and diagnostic indicators is next-generation sequencing. Coexisting mutations are associated with reduced response to therapy, shortened overall survival, and a higher risk of transformation to acute myeloid leukemia or myelofibrosis. The study group consisted of 42 patients with the diagnosis of *BCR::ABL1*-negative MPN and the presence of a mutation in the *CALR* gene. The research material was archival, and DNA was obtained from patients’ peripheral blood. Forty genes (17 genes, 23 hotspots) were sequenced using the commercial kit AmpliSeq for Illumina Myeloid Panel applying the targeted next-generation sequencing approach. For the study, the Illumina MiniSeq platform was used. The analysis of the obtained genetic results was carried out using bioinformatics tools and genetic databases. We studied 42 *CALR*-positive ET (*n* = 28) and MF (*n* = 14) patients with NGS panel testing. The median age at diagnosis of the entire patient series was 58 years. Additional mutations were detected in 48% of patients in the whole cohort. The most frequently mutated genes in the study population were *ASXL1*, *TET2*, and *DNMT3A*, which are largely associated with epigenetic regulatory mechanisms. NGS panel studies represent a breakthrough in the diagnostic and prognostic evaluation of MPNs with *CALR* mutations. The ability to perform such a comprehensive study provides valuable information on the biology of the disease and the selection of the appropriate treatment regimen. The use of new technologies shows that not only driver mutations have clinical significance for the patient. NGS has the potential to increase the precision and effectiveness of diagnosis and prognosis.

## Background

The classical *BCR::ABL1* negative myeloproliferative neoplasms are a group of blood disorders characterized by the overproduction of mature blood cells in the bone marrow. This group of diseases includes polycythemia vera, essential thrombocythemia, and myelofibrosis. “The Fifth edition of the World Health Organization Classification of Haematolymphoid Tumours: Myeloid and Histiocytic/Dendritic Neoplasms” and the International Consensus Classification (ICC) of Myeloid Neoplasms and Acute Leukemias provide the current criteria for the diagnosis of myeloproliferative neoplasms. The clinical picture, laboratory results, analysis of the bone marrow biopsy, and genetic testing for “driver mutations” in genes *JAK2*, *CALR*, or *MPL* are used to make the diagnosis. In addition to the fact that these mutations are crucial for diagnosis, they also have prognostic and therapeutic significance (Khoury et al. 2022) (Arber et al. 2022). Approximately 95–98% of patients with PV and 50–60% of patients with ET and PMF have the *JAK2V617F* mutation. Rare insertions or deletions in exon 12 of the *JAK2* gene may be present in 2–3% of PV patients who are *JAKV617F*-negative. Mutations in the *JAK2* gene cause uncontrolled cell proliferation and constitutive activation of the STAT signaling pathway (Tifferi 2021) (Tefferi and Barbui 2020;). Approximately 20–25% of ET cases and 35% of PMF cases have CALR mutations. Mutations in this gene are not found in patients with PV. Research has demonstrated that CALR mutations can activate the JAK-STAT signaling pathway by activating the thrombopoietin receptor MPL (Chachoua et al. 2016). One to 4% of patients with essential thrombocythemia and 5–10% of all myelofibrosis patients have *MPL* mutations. *MPL* mutations activate *JAK2* and the thrombopoietin pathway (Guglielmelli et al. 2021). Nevertheless, driver mutations are not the only mutations identified in patients with MPNs. The incredible progress that has been made over the last 10 years in the field of genetic diagnostics has prompted the identification of additional genetic abnormalities linked to MPN. Next-generation sequencing (NGS) has become widely used as a result of technological advances in molecular techniques. In light of new WHO and ICC recommendations, NGS plays a very important role in hematooncology diagnostics. Therefore, the availability of NGS for patients with blood malignancy is expanding. The most commonly used method is gene panel sequencing ( Loscocco et al. 2020) (Tefferi et al. 2016a,b) (Tefferi 2023) (Barbui et al. 2018).

We retrospectively studied a cohort of 42 *CALR*-mutated MPN patients by a targeted NGS panel of 40 genes. We analyzed the impact of mutations on clinical outcomes and disease progression.

## Methods

**Fig. 1:**
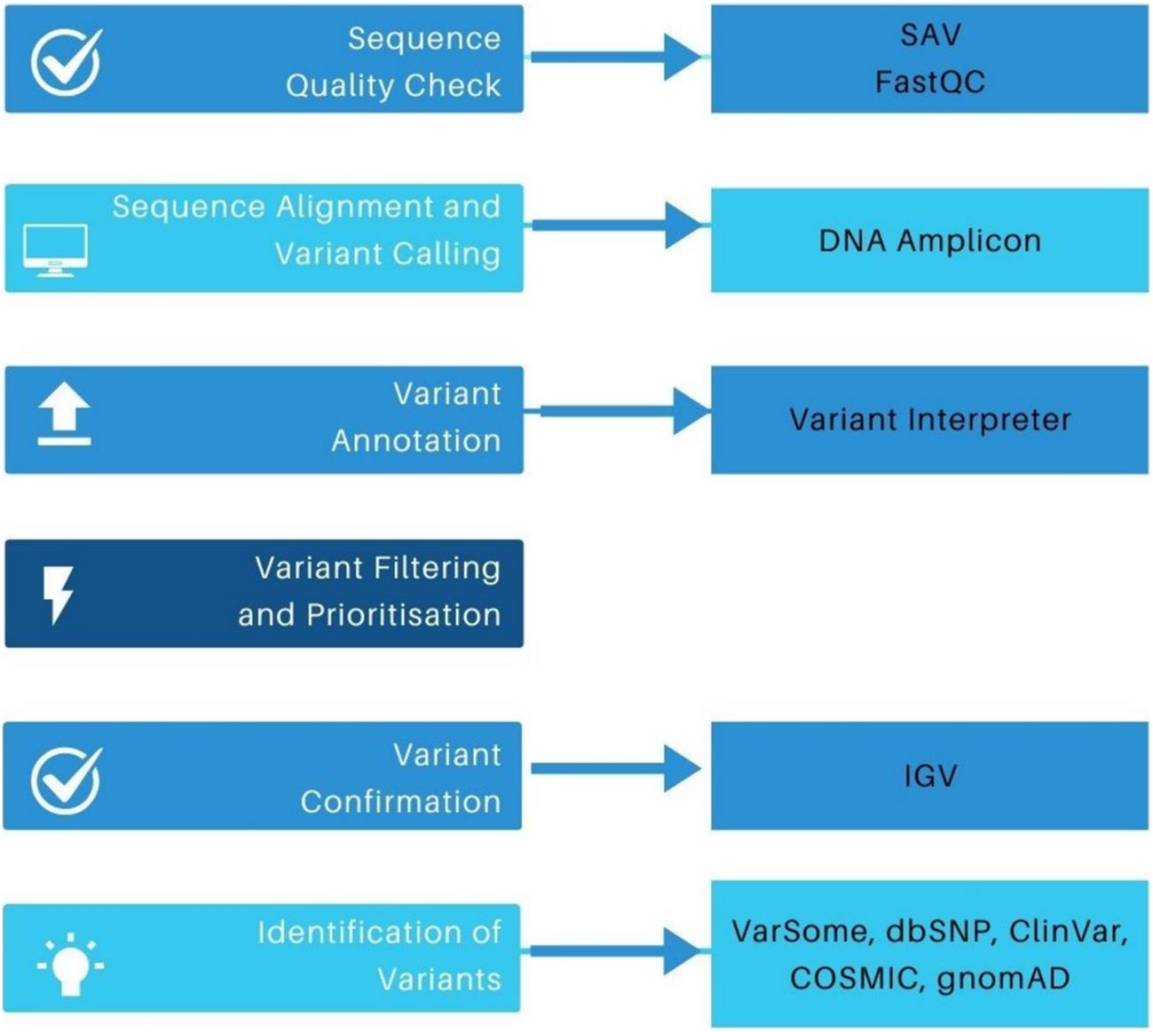
Schematic representation of the steps for analyzing NGS sequencing data

We studied 42 *CALR*-mutated MPN patients (28 ET and 14 MF) with a median follow-up of 4 years (1–16). Patients were diagnosed in our department between 2015 and 2020; genetic samples had been obtained at the time of molecular diagnosis. Patients diagnosed in 2015 were officially classified according to the WHO 2008 classification, but with the implementation of the WHO 2016 classification, their diagnoses were updated, especially in the case of new molecular tests, such as mutation in the CALR gene. Patients diagnosed between 2016 and 2020 were classified according to the WHO 2016. NGS testing was performed at different time points. In some patients, NGS was performed at the beginning of the diagnosis, in others during complementary molecular testing. The first study to detect the type of mutation in the *CALR* gene was performed by PCR fragment analysis. NGS testing was performed retrospectively on each patient on DNA material from the time of molecular diagnosis. All of 17 full genes (*ASXL1*, *BCOR*, *CALR*, *CEBPA*, *ETV6*, *EZH2*, *IKZF1*, *NF1*, *PHF6*, *PRPF8*, *RB1*, *RUNX1*, *SH2B3*, *STAG2*, *TET2*, *TP53*, *ZRSR2*) and 23 hotspot genes (*ABL1*, *BRAF*, *CBL*, *CSF3R*, *DNMT3A*, *FLT3*, *GATA2*, *HRAS*, *IDH1*, *IDH2*, *JAK2*, *KIT*, *KRAS*, *MPL*, *MYD88*, *NPM1*, *NRAS*, *PTPN11*, *SETBP1*, *SF3B1*, *SRSF2*, *U2AF1*, *WT1*) were covered by an Ampliseq™ for Illumina Myeloid Panel (Illumina, San Diego, USA). The NGS panel included the CALR gene; therefore, the type of CALR mutation was confirmed in all patients selected for the study. Library preparation and sequencing using the MiniSeq platform (Illumina) were performed according to the manufacturer’s instructions. It was a targeted panel for investigating somatic variants associated with hematological malignancies. This panel allows the detection of changes in the type of substitution, insertion, and deletion of several nucleotides. The processes involved in the quality control and bioinformatics analysis of the NGS data are schematically represented in Fig. 1. With the Illumina Sequence Analysis Viewer program, the sequencing metrics were displayed. Using the FastQC software on the Illumina BaseSpace™ Sequence Hub, the raw NGS data’s quality was evaluated. The sequencing data was analyzed using a sequence alignment (DNA Amplicon) and variant calling applications (Variant Interpreter) also on the Illumina BaseSpace™ Sequence Hub. Additionally, a comprehensive report summarizing amplicon data, coverage by amplicon area, base-level statistics, and sample read quality was generated by the DNA Amplicon app. For mutation calling, the minimum coverage of called regions was set to 500 and the minimum variant allele frequency (VAF) was set to 3%. The sequencing data was aligned against the Genome Reference Consortium human genome build 37 (GRCh37). Using the Integrative Genomics Viewer (IGV) program (Broad Institute, Cambridge, USA), aligned read (.bam) files for each sample were manually examined to verify the existence of the filtered-in and prioritized variants. To identify variants, available genetic databases such as VarSome Clinical, COSMIC, dbSNP, ClinVar, gnomAD, and publications were used. Variants were categorized as pathogenic, likely pathogenic, and variant of uncertain significance (VUS).

## Statistical analysis

The two-sample *t*-test was used to compare numerical variables, and the chi-square or Fisher’s exact test was used to compare categorical variables. The association between the CALR mutant burden and other laboratory findings or age at diagnosis was assessed using correlation analysis. A *p*-value of less than 0.05 was deemed statistically significant. The Statistica software (TIBCO Software Inc.) was used for conducting statistical analysis.

## Results

**Fig. 2:**
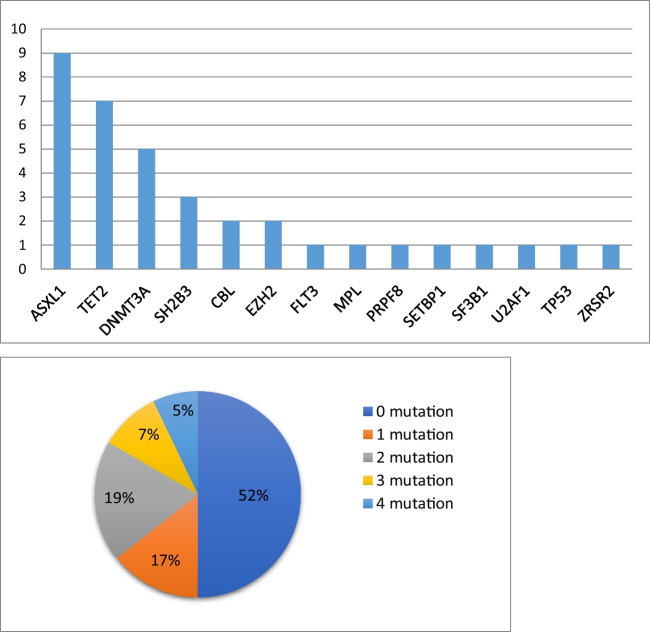
Distribution of additional mutations

This study was approved by Ethics committee of Wroclaw Medical University. Forty-two *CALR*-positive ET (*n* = 28) and MF (*n* = 14) patients underwent targeted sequencing. The median age at diagnosis of the entire patient series was 58 years (range, 24–87); 20 were males (47.6%). Due to the fact that the research material was archival and came from the years 2015–2020, in Table 1, we present clinical and biological information about patients for whom we were able to obtain a medical history. Each group was further divided according to the type of CALR mutation. In the study group, we had patients only with a 52-bp deletion (type-1 mutation) and a 5-bp insertion (type-2 mutation) (Appendix 1). Type 1 *CALR* mutation occurred in 54% (*n* = 15), and type 2 occurred in 46% (*n* = 13) of patients with ET. In MF, type 1 definitely predominates and occurred in 93% (*n* = 13) of cases, only one patient had a type 2 mutation. Additional mutations were detected by the NGS sequencing panel in 48% of patients in the whole cohort (Appendix 2). The analysis detected 44 pathogenic, likely pathogenic, or uncertain significance variants. According to scientific reports, we reported variants on VAF above 3%. The most frequently mutated genes in the study population were *ASXL1*, *TET2*, and *DNMT3A*, which are largely associated with epigenetic regulatory mechanisms (Fig. 2). Twelve variants were detected in the *ASXL1* gene, including one patient with 2 co-occurrent variants, and one patient with three co-occurrent variants. Fourteen patients had two or more than two additional mutations (33%). Mutations in genes: *ASXL1*, *SRSF2*, *EHZ2*, *IDH1*, *IDH2*, *U2AF1*, defined as high molecular risk mutations (HRM) had ten patients. Seven patients transformed from ET to MF. Among these patients, five had additional mutations. One patient with four additional mutations, including two co-occurrent variants in the *DNMT3A* gene, mutation in the *TP53* gene and *U2AF1* gene, transformed from ET to MF and then from MF to AML. After alloHSCT, the patient had a relapse and died. Two patients diagnosed with myelofibrosis transformed into AML, both patients had additional mutations.

**Table 1:** Clinical characteristics of ET and MF patients stratified according to the CALR mutant type. Asterisk (*) symbol indicates data obtained for all patients in the study group

	ET patients		MF patients
	CALR type I mutation (*n* = 14)	CALR type II mutation (*n* = 12)	CALR type I mutation (n = 13)
Age at diagnosis, median (range)	59.5 (24–78)	53 (34–60)	63 (33–87)
Sex, male/female	6/8	3/7	7/5
Leucocytosis at diagnosis (10^9^/l), median (range)	9 (5.5–16.8)	6.4 (4.2–10.1)	8.4 (3.8–18.9)
Hemoglobin at diagnosis (g/dl), median (range)	13.5 (7.2–14.7)	13.6 (11.3–15.6)	11.5 (8.4–14.2)
Platelets at diagnosis (10^9^/l), median (range)	708 (386–881)	804 (500–1400)	616 (251–1691)
Splenomegaly	4 (28.6%)	3 (25%)	8 (61.5%)
IPSET/MIPSS70 risk groups			
Low risk	12	10	8
Intermediate risk	2	0	3
High risk	0	0	2
Allele burden of CALR, median, (range)	34.8% (11.6–53)*	36.2% (9.3–72)*	39.9% (12–52.1)*
Thrombosis before/at diagnosis	0/1	1/1	1/0
Additional mutations	4*	9*	7*

For further analysis, due to the NGS test being performed at different time points, patients were divided into two groups. The first group included patients who underwent NGS during initial diagnosis or before the implementation of cytoreductive treatment (*n* = 31). The second group included those who underwent the test during later genetic diagnostics, after the introduction of methods detecting mutations in the *CALR* gene or during disease progression (*n* = 11).

For Group 1 (*n* = 31), follow-up data were available for 28 patients, ranging from 5 to 195 months with a median follow-up time of 49 months. The median *CALR* mutation allele burden in the first group was 36.2% (9.3–85.5%). To estimate allele burden, we used VAF detected by NGS. A moderate correlation was found between age at diagnosis and *CALR* allele burden. There was no difference between *CALR* allele burden and sex or diagnosis of ET or MF. Information on splenomegaly was available in 24 patients. We observed that patients with splenomegaly had a higher allele burden (*p* = 0.0008).

We observed that high *CALR* mutation burden correlated positively with leukocyte count, whereas it correlated negatively with hemoglobin level and weakly negatively with platelet count.

Moreover, we were able to obtain information on disease transformation for 25 patients. In 5 patients, transformation to MF or AML occurred. The *CALR* mutation burden was significantly higher among patients who experienced disease transformation (*p* = 0.001). Patients who died shortly after diagnosis, median 15 months (range 5–56), had significantly higher allele burden than patients who survived to the end of follow-up (*p* = 0.0002).

In this group, additional mutations had 45.2% of patients. We observed that the *CALR* allele burden was slightly higher in patients with additional mutations, but without statistical significance (*p* = 0.096). We observed that co-occurring mutations were more common in older patients (*p* = 0.002).

The study group size was too small to determine whether the presence of additional mutations influenced disease transformation to MF or AML. Although we observed that among 5 patients who had disease transformation, four patients had additional mutations. Two of them had mutations from the HRM group.

Only one patient had thromboembolic or hemorrhagic episodes before diagnosis, but we were unable to obtain information on the type of episodes. He had an additional mutation in the *TET2* gene. One patient had an ischemic stroke after diagnosis and had no additional mutations. For three patients, the medical history did not contain information about thromboembolic or hemorrhagic episodes. The remaining patients did not report any episodes.

The second group was much smaller and consisted of 11 patients diagnosed only with ET. The NGS panel testing in this group was conducted several years after diagnosis. Median time 11 years (range, 4–24). For Group 2, follow-up ranged from 72 to 315 months with a median follow-up time of 208 months. The median *CALR* mutation allele burden was 40.8% (30.5–72%). There was no difference between *CALR* allele burden and sex. We also observed that *CALR* allele burden was not associated with the occurrence of additional mutations (*p* = 0.202).

In six patients, transformation to MF or AML occurred. In this group, the *CALR* mutation burden was not significantly higher among patients who experienced disease transformation (*p* = 0.230). It should be emphasized that the NGS test in this group was performed several years after the diagnosis was made. Along with the progression of the disease, the burden allele could also increase.

In this group, additional mutations were observed in 54.55% of patients. Among this group were six patients who transformed to MF or AML. Five of them had additional mutations. Four patients had mutations from the HRM group. Only one patient had thromboembolic or hemorrhagic episodes after diagnosis. The patient had an ischemic stroke after aortic valve replacement. This patient had a *CALR* type II mutation and two additional mutations in the MPL and *SH2B3* genes. Both genes had variants of uncertain significance.

One of the limitations of our study is that the NGS testing was done at different time points. In some patients, NGS was performed at the beginning of the diagnosis; in others, it was performed during complementary molecular tests to search for mutations in the *CALR* gene after the method assessing the presence of mutations in this gene was introduced. However, the former limitation is somewhat offset by the fact that the majority of patients with *CALR* mutations in our cohort had NGS testing carried out before the first intervention/treatment. Among the 42 patients, 10 (23,8%) died, 32 (76,2%) survived, and 8 (19%) were out of contact for follow-up.

## Discussion

We studied 42 patients with MPN and *CALR* mutations. We performed gene panel sequencing in all patients. Over the past decades, NGS has become an important tool in the field of oncology and a very useful tool in the management of myeloproliferative neoplasms. Genome profiling provides the basis for a personalized prognosis prediction for each patient. Over the past decades, significant progress has been made in our understanding of the pathophysiology of *BCR::ABL1*-negative myeloproliferative neoplasms. *CALR* mutations, characteristic of essential thrombocythemia and primary myelofibrosis, represent a significant group of MPN cases and, compared with other mutations such as *JAK2* or *MPL*, show distinct clinical and prognostic features. The greatest advantage of NGS panel testing is that we can simultaneously detect mutations in multiple genes and quantify mutation burden. The comprehensive data obtained from NGS can significantly impact clinical decision-making. Most mutations in the *CALR* gene are classified as type 1 or type 2. Type 1 is slightly more common (55–61%) among ET patients, whereas type 2 occurs in approximately 38% of cases (Gangat et al. 2024) ( Loscocco et al. 2024). Nevertheless, in PMF, type 1 clearly predominates and appears in about 70% of cases, while type 2 only appears in 13% of cases (Tefferi et al. 2018). In our study group, the incidence of *CARL* type 1 or 2 mutations was similar to those reported in the literature. Access to information regarding the allele burden of *CALR* mutations is limited. Among ET patients, high *CALR* mutant burden has been linked to increased leukocyte and platelet counts, but decreased hemoglobin levels. Patients with ET and splenomegaly presented a higher level of LDH or higher rate of myelofibrotic transformation, which was associated with a higher *CALR* allele burden (Gángó et al. 2018) (Andrikovics et al. 2014). In patients with MF, it was noted that there was no significant difference between allele burden and sex, age, leukocyte count, splenomegaly, thrombosis, or bleeding. Statistically significant lower platelet and Hb concentration, higher peripheral blood CD34 + cell count, and the need for cytoreductive therapy were found in patients with high *CALR* allele burden. A trend towards a higher incidence of high-risk mutations was observed in patients with high *CALR* VAF. *ASXL1* mutations were significantly more frequent in patients with high *CALR* allele burden (Guglielmelli et al. 2023). In our patients from the first group, we had similar observations regarding laboratory tests, except for the higher platelet count. And similar results regarding *CALR* allele burden and splenomegaly. There have been reports of increased *CALR* allele burden associated with disease progression (Cottin et al. 2020) (Aubin et al. 2024) (Cavalloni et al. 2017). Our findings support this concept. In the first group, the *CALR* mutation burden was significantly higher in patients who experienced disease transformation (*p* = 0.001). Additionally, patients who died shortly after diagnosis, median 15 months (range 5–56), had significantly higher allele burden than patients who survived to the end of the follow-up period (*p* = 0.0002). The authors also indicate the existence of a correlation between increased *CALR* allele burden and the introduction of cytoreductive therapy. However, the results are not consistent. It is interesting to note that type I *CALR* mutations in MF are linked to longer survival and reduced risk of disease transformation and may potentially reduce the negative effects of *ASXL1* and *SRSF2* mutations, which are usually associated with worse prognosis (Prins et al. 2020) (Guglielmelli et al. 2015) (N. M. Tefferi et al. 2018) (Tefferi et al. 2014). Whereas in patients with ET, *CALR* type I is associated with an increased risk of transformation. Another study noted that the time to MF progression is significantly shorter in *CALR* type I than in type II mutation (Prins et al. 2020) (Rotunno et al. 2016). We have not observed such an association in our cohort, probably due to the small number of patients. Molecular testing, particularly next-generation sequencing (NGS), is now widely available in clinical laboratories, and as a result, the paradigms for myeloid neoplasm diagnosis and classification have changed. More than 50% of patients with PV/ET and 80% of PMF cases had additional mutations. However, these mutations occur in all hematological malignancies and are not specific to MPNs. These additional mutations usually affect genes involved in epigenetic regulation (*TET2*, *DNMT3A*, *IDH1/2*, *ASXL1*, and *EZH2*), mRNA splicing (*SF3B1*, *SRSF2*, *U2AF1*, and *ZRSR2*), signaling pathways (*NRAS*, *KRAS*, *CBL*, *NF1*, *SH2B3*, and *PTPN11*), and transcription factors (*RUNX1* and *TP53*). Additional mutations are associated with an increased risk of disease transformation and reduced overall survival. (Tefferi et al. 2016a) (b) (Luque et al. 2017) (Yan et al. 2022 ) (Luque Paz et al. 2023 ) (V. 2002 ). We have found additional mutations in 48% of the patients in the entire group. *ASXL1*, *TET2*, and *DNMT3A* were the most frequently mutated genes; these genes are mostly linked to epigenetic regulatory processes. Nevertheless, it was not possible to determine if the existence of additional mutations affected the disease’s transformation to MF or AML due to the limited size of the study group. Despite this, ten patients had mutations from the HRM group. Seven patients transformed from ET to MF. Five of these patients had additional mutations. A patient who transformed from ET to MF and subsequently from MF to AML had four additional mutations. Two individuals with myelofibrosis developed AML both had extra mutations. Also, in our first group, the occurrence of additional mutations was associated with older age. In the second group, the percentage of additional mutations was higher than in the first one, most likely because the NGS test was performed several years after diagnosis. Additional mutations may increase with the patient’s age and disease progression. In general, *CALR* mutations have been linked to a decreased risk of thrombosis (Lewandowski 2021, (Tefferi 2023). Only three patients in the entire group had thromboembolic or hemorrhagic episodes. A notable limitation of our study is the combination of patients with ET and PMF in the first group in the analysis of CALR mutations and co-occurring mutations. We recognize that these diseases differ in clinical course and molecular profiles, which may influence the interpretation of our findings. Given these limitations, we decided to combine the two groups in the primary analysis. This approach increased statistical power and allowed for more interpretable results while still capturing the common role of CALR mutations in the pathogenesis of myeloproliferative neoplasms. However, we acknowledge that this decision may have masked disease-specific differences. Future studies with larger cohorts should aim to evaluate CALR mutations and co-occurring mutations in ET and PMF separately to better understand their distinct roles in these diseases.

## Conclusions

The identification of myeloproliferative neoplasms has been improved with the use of the NGS method. Further research and development in this area promises to improve patient outcomes through more precise and tailored therapeutic approaches. Prognostic factors can be identified and disease categorization systems can be developed further with the use of sequencing data and mathematical algorithms. For instance, the existence of particular co-mutations may point to a patient’s vulnerability to a particular inhibitor or point to the advantages of combination treatments. Furthermore, to track clonal evolution and therapy response, longitudinal NGS studies are necessary. This could result in more tailored and dynamic care plans for patients with *CALR* mutation MPNs. NGS panel testing has many advantages, yet there are limitations when using it in clinical settings. Highly trained staff and strong bioinformatics support are needed for the interpretation of complicated NGS data. Variants of uncertain significance (VUS) remain a problem and ongoing database updates and functional studies are needed to clarify their significance.

## Data Availability

The datasets generated during the current study are available in the Sequence Read Archive (SRA) repository, accession number: PRJNA1168192.

## Appendix 1

**Table:**

Lp	Nucleotide ID	Protein ID	Type	Mutation	COSMIC
1	c.1099_1150del	p.Leu367ThrfsTer46	type-1	deletion	COSV57116546
2	c.1154_1155insTTGTC	p.Lys385AsnfsTer47	type-2	insertion	COSV57116551
3	c.1154_1155insTTGTC	p.Lys385AsnfsTer47	type-2	insertion	COSV57116551
4	c.1155_1156insTGTCG	p.Glu386CysfsTer46	type-2	insertion	COSV57117852
5	c.1154_1155insTTGTC	p.Lys385AsnfsTer47	type-2	insertion	COSV57116551
6	c.1099_1150del	p.Leu367ThrfsTer46	type-1	deletion	COSV57116546
7	c.1099_1150del	p.Leu367ThrfsTer46	type-1	deletion	COSV57116546
8	c.1099_1150del	p.Leu367ThrfsTer46	type-1	deletion	COSV57116546
9	c.1099_1150del	p.Leu367ThrfsTer46	type-1	deletion	COSV57116546
10	c.1099_1150del	p.Leu367ThrfsTer46	type-1	deletion	COSV57116546
11	c.1154_1155insTTGTC	p.Lys385AsnfsTer47	type-2	insertion	COSV57116551
12	c.1099_1150del	p.Leu367ThrfsTer46	type-1	deletion	COSV57116546
13	c.1099_1150del	p.Leu367ThrfsTer46	type-1	deletion	COSV57116546
14	c.1099_1150del	p.Leu367ThrfsTer46	type-1	deletion	COSV57116546
15	c.1099_1150del	p.Leu367ThrfsTer46	type-1	deletion	COSV57116546
16	c.1154_1155insTTGTC	p.Lys385AsnfsTer47	type-2	insertion	COSV57116551
17	c.1154_1155insTTGTC	p.Lys385AsnfsTer47	type-2	insertion	COSV57116551
18	c.1099_1150del	p.Leu367ThrfsTer46	type-1	deletion	COSV57116546
19	c.1154_1155insTTGTC	p.Lys385AsnfsTer47	type-2	insertion	COSV57116551
20	c.1099_1150del	p.Leu367ThrfsTer46	type-1	deletion	COSV57116546
21	c.1099_1150del	p.Leu367ThrfsTer46	type-1	deletion	COSV57116546
22	c.1099_1150del	p.Leu367ThrfsTer46	type-1	deletion	COSV57116546
23	c.1099_1150del	p.Leu367ThrfsTer46	type-1	deletion	COSV57116546
24	c.1103_1154del	p.Leu367ThrfsTer46	type-1	deletion	COSV57116546
25	c.1099_1150del	p.Leu367ThrfsTer46	type-1	deletion	COSV57116546
26	c.1099_1150del	p.Leu367ThrfsTer46	type-1	deletion	COSV57116546
27	c.1099_1150del	p.Leu367ThrfsTer46	type-1	deletion	COSV57116546
28	c.1099_1150del	p.Leu367ThrfsTer46	type-1	deletion	COSV57116546
29	c.1099_1150del	p.Leu367ThrfsTer46	type-1	deletion	COSV57116546
30	c.1154_1155insTTGTC	p.Lys385AsnfsTer47	type-2	insertion	COSV57116551
31	c.1099_1150del	p.Leu367ThrfsTer46	type-1	deletion	COSV57116546
32	c.1099_1150del	p.Leu367ThrfsTer46	type-1	deletion	COSV57116546
33	c.1154_1155insTTGTC	p.Lys385AsnfsTer47	type-2	insertion	COSV57116551
34	c.1099_1150del	p.Leu367ThrfsTer46	type-1	deletion	COSV57116546
35	c.1154_1155insTTGTC	p.Lys385AsnfsTer47	type-2	insertion	COSV57116551
36	c.1099_1150del	p.Leu367ThrfsTer46	type-1	deletion	COSV57116546
37	c.1154_1155insTTGTC	p.Lys385AsnfsTer47	type-2	insertion	COSV57116551
38	c.1154_1155insTTGTC	p.Lys385AsnfsTer47	type-2	insertion	COSV57116551
39	c.1154_1155insTTGTC	p.Lys385AsnfsTer47	type-2	insertion	COSV57116551
40	c.1099_1150del	p.Leu367ThrfsTer46	type-1	deletion	COSV57116546
41	c.1099_1150del	p.Leu367ThrfsTer46	type-1	deletion	COSV57116546
42	c.1099_1150del	p.Leu367ThrfsTer46	type-1	deletion	COSV57116546

## Appendix 2

**Table:**

Lp	Gene (additional mutation)	Nucleotide ID	VAF %	Protein ID	Variant classification	COSMIC
**1223/20**	ASXL1	c.1249C>T	3,5	p.Arg417Ter	Pathogenic	COSV60102853
**1658/20**	ASXL1	c.1900_1922del	16	p.Glu635ArgfsTer15	Pathogenic	COSV60102280
	ASXL1	c.1917_1927del	14	p.Ala640GlyfsTer14	Likely pathogenic	COSV104623419
	ASXL1	c.1438G>T	4,3	p.Glu480Ter	VUS	COSV60104059
	EZH2	c.1987_1990del	23,4	p.Tyr663IlefsTer11	Pathogenic	NA
**1755/20**	CBL	c.1111T>A	3,5	p.Tyr371Asn	Pathogenic	COSV50630502
	ASXL1	c.1782C>A	41,1	p.Cys594Ter	Likely pathogenic	COSV60106156
	EZH2	c.170del	43	p.Asn57ThrfsTer15	Pathogenic	NA
**223/19**	TET2	c.3782G>A	45,9	p.Arg1261His	Pathogenic	COSV54396706
**1739/19**	DNMT3A	c.2644C>T	45,3	p.Arg882Cys	Pathogenic	COSV53036332
	SETBP1	c.2608G>A	4,1	p.Gly870Ser	Pathogenic	COSV56311834
**59/19**	ASXL1	c.1772dup	31,6	p.Tyr591Ter	Pathogenic	COSV60103309
	DNMT3A	c.1906G>A	6,5	p.Val636Met	Pathogenic	COSV53039866
	PRPF8	c.4781G>A	32,1	p.Cys1594Tyr	VUS	NA
	SH2B3	c.232G>A	52,8	p.Glu78Lys	VUS	COSV57983338
**82/19**	DNMT3A	c.2204A>G	25,1	p.Tyr735Cys	Likely pathogenic	COSV53036596
**112/19**	SF3B1	c.1998G>T	46,4	p.Lys666Asn	Pathogenic	COSV59205799
**550/18**	DNMT3A	c.2185C>T	6,8	p.Arg729Trp	Likely pathogenic	COSV53036577
**851/18**	ASXL1	c.2583dup	21,2	p.Phe862IlefsTer2	Likely pathogenic	NA
	IDH2	c.419G>A	4,8	p.Arg140Gln	Pathogenic	COSV57468751
**1180/18**	ASXL1	c.665A>C	53,7	p.Glu222Ala	VUS	NA
	TET2	c.5618T>C	34,9	p.Ile1873Thr	Pathogenic	COSV54396330
	TET2	c.5059C>T	14,5	p.Gln1687Ter	Likely pathogenic	COSV54399904
**15085/17**	TET2	c.5618T>C	82,2	p.Ile1873Thr	Pathogenic	COSV54396330
**15492/17**	FLT3	c.1634A>G	24,8	p.Tyr545Cys	VUS	COSV105839707
	SH2B3	c.1454_1477del	51,5	p.Asp485_Trp492del	Likely pathogenic	COSV57982301
	TET2	c.3532del	25	p.Glu1178LysfsTer48	Pathogenic	COSV54397755
**14833/17**	ASXL1	c.2338C>T	31,9	p.Gln780Ter	Pathogenic	COSV60107863
	ASXL1	c.2464dup	6,2	p.Thr822AsnfsTer11	Likely pathogenic	COSV60102747
**11511/15**	ASXL1	c.1974_1978del	46,5	p.Gly659fs	Likely pathogenic	NA
	CBL	c.1149A>G	42	p.Ile383Met	VUS	COSV50634832
**11524/15**	ZRSR2	c.868C>T	83,8	p.Arg290*	Likely pathogenic	COSV57067798
	TET2	c.2562del	15	p.Phe854LeufsTer19	Likely pathogenic	NA
**12641/16**	TET2	c.3811del	28,2	p.Cys1271AlafsTer92	Likely pathogenic	NA
	TET2	c.3505G>A	27	p.Gly1169Arg	VUS	COSV54425134
**14746/17**	DNMT3A	c.2628C>G	38,2	p.Asp876Glu	VUS	NA
	DNMT3A	c.2111T>C	40,5	p.Val704Ala	VUS	NA
	U2AF1	c.101C>T	13,4	p.Ser34Phe	Pathogenic	COSV52341059
	TP53	c.659A>G	31,3	p.Tyr220Cys	Pathogenic	COSV52661282
**14815/17**	ASXL1	c.2278C>T	38,6	p.Gln760*	Likely pathogenic	COSV60106248
	TET2	c.1061C>G	40,7	p.Ser354Ter	Pathogenic	COSV54398893
**10388/15**	SH2B3	c.232G>T	3,8	p.Glu78Lys	VUS	COSV57983338
	SH2B3	c.1145G>C	49,6	p.Gly382Ala	VUS	NA
	MPL	c.1502T>C	42,4	p.Val501Ala	VUS	COSV65244340

## Abbreviations

ET: Essential thrombocythemia
MF: Myelofibrosis
MPN: Myeloproliferative neoplasms
AML: Acute myeloid leukemia
NGS: Next-generation sequencing
ICC: International Consensus Classification
VAF: Variant allele frequency
